# To Cure a Cold

**Published:** 1855-05

**Authors:** 


### TO CURE A COLD.

A bad cold, like measles or mumps, or other similar ailments, will run its course of about ten days, in spite of what may be done for it, unless remedial means are employed within forty-eight hours of its inception. Many a useful life may be spared to be increasingly useful, by cutting a cold short off, in the following safe and simple manner. On the first day of taking a cold, there is a very unpleasant sensation of chilliness. The moment you observe this, go to your room and *stay there;* keep it at such a temperature as will entirely prevent this chilly feeling, even if it requires a hundred degrees of Fahrenheit. In addition, put your feet in water, half leg deep, as hot as you can bear it, adding hotter water from time to time for a quarter of an hour, so that the water shall be hotter when you take your feet out than when you put them in; then dry them thoroughly, and put on warm thick woolen stockings, even if it be summer, for summer colds are the most dangerous; and for twenty-four hours, eat not an atom of food; but drink as largely as you desire of any kind of warm teas, and at the end of that time, if not sooner, the cold will be effectually broken, without any medicine whatever.

Efficient as the above means are, not one in a thousand will attend to them, led on as men are by the hope that a cold will pass off of itself; nevertheless this article will now and then pass under the eye of a wise man, who does not choose to run the double risk of taking physic and dying too.

